# Incidence, prevalence, and comorbidities of juvenile idiopathic arthritis in Germany: a retrospective observational cohort health claims database study

**DOI:** 10.1186/s12969-022-00755-x

**Published:** 2022-11-16

**Authors:** Gerd Horneff, Julia Borchert, Ria Heinrich, Simon Kock, Pascal Klaus, Heike Dally, Christine Hagemann, Joanna Diesing, Tonio Schönfelder

**Affiliations:** 1 Department of General Paediatrics, Asklepios Clinic Sankt Augustin, Sankt Augustin, Germany; 2 Department of Paediatric and Adolescents Medicine, University Hospital of Cologne, Cologne, Germany; 3 Scientific Institute for Health Economics and Health System Research (WIG2 GmbH), Leipzig, Germany; 4 Institute for Applied Health Research (InGef), Berlin, Germany; 5 Pfizer Pharma GmbH, Berlin, Germany; 6 Lehrstuhl Gesundheitswissenschaften/ Public Health, Technische Universität Dresden, Dresden, Germany

**Keywords:** Prevalence, Incidence, Comorbidities, Juvenile idiopathic arthritis, JIA, Polyarticular JIA, Burden of disease

## Abstract

**Background:**

Juvenile idiopathic arthritis (JIA) describes heterogenous categories of chronic inflammatory rheumatic conditions of unknown origin in children and adolescents. Epidemiological data in the literature vary, depending on geographic location, ethnicity and the case definition used. We evaluated epidemiology, especially that of the categories defined by the International League of Associations for Rheumatology (ILAR).

**Methods:**

Using data from two different longitudinal health claims databases (WIG2 and InGef) from January 1^st^, 2013 to December 31^st^, 2019, we looked at patients aged 2 to 15 years old with at least one main inpatient or two secondary inpatient/verified outpatient ICD-10 diagnoses in at least two different quarters within one calendar year. We calculated prevalence and incidence (per 100,000 patients) and extrapolated data to the entire German population, looking at differences in gender and age groups. Additionally, we collected data on “other” not necessary comorbidities in our JIA patient population.

**Results:**

Of the 3–4 million patients in the databases (respectively) in 2018, we found a total of 546 (WIG2) and 849 (InGef) patients that met our JIA case definition, with an incidence of 34 (29–41) and 60 (53–67) and prevalence of 133 (122–145) and 168 (157–179). Both incidence and prevalence throughout the age range were mostly higher in females than males, however the difference between females and males increased with increasing age. Of the ILAR categories, oligoarthritis was the most prevalent (70 and 91 per 100,000), with about half of our JIA patients in this category, followed by undifferentiated arthritis (49 and 56 cases per 100,000) and rheumatoid factor negative (RF-) (31 and 39 per 100,000). Incidence in 2018 was the highest in these three categories. Atopic dermatitis, vasomotor and allergic rhinitis, and uveitis were the pre-defined comorbidities seen most often in both databases.

**Conclusion:**

This study provides current incidence and prevalence JIA data in Germany, contributing to knowledge on burden of disease and tools for healthcare planning.

**Supplementary Information:**

The online version contains supplementary material available at 10.1186/s12969-022-00755-x.

## Background

Juvenile idiopathic arthritis (JIA) describes heterogenous categories of chronic inflammatory rheumatic conditions of unknown origin. Affecting children and adolescents [1], onset occurs before the age of 16 years and persists at least 6 weeks, and is concluded following exclusion of any other known arthritis causes [2]. JIA pathogenesis is suspected as a result of genetic predisposition, immune-related factors and environmental influence, however specific agents have not been identified [3].

Depending on the JIA category, patients may show prominent systemic symptoms, such as fever, rash, and serositis (as in systemic arthritis), or have arthritic involvement of few or numerous joints [2]. More severe manifestations include general growth retardation, accelerated growth of an affected joint or erosive joint destruction more common in rheumatoid factor positive (RF +) disease and an increased risk of infection and malignancy, as well as comorbidities including second autoimmune diseases [4–6]. The goal of JIA therapy is to reduce inflammation and, ideally, to achieve remission (inactive disease). This typically includes a multiple pharmaco-medical treatment with non-steroidal anti-inflammatory drugs (NSAIDs), glucocorticoids (GCs), conventional synthetic disease-modifying anti-rheumatic drugs (csDMARDs), and biological disease-modifying anti-rheumatic drugs (bDMARDs) as well as targeted small molecules such as the Janus kinase (JAK) inhibitors [2].

The latest revision of the criteria describing JIA in 2001 by the International League of Associations for Rheumatology (ILAR) [2] modified existing criteria [7], and described seven categories falling under the term JIA. These define homogenous and mutually exclusive groups to facilitate communication among physicians and researchers. There are six categories defined by how many joints are involved, by extraarticular manifestations, and by the presence of certain markers [8]; systemic arthritis (sJIA), oligoarthritis (oligoJIA), rheumatoid factor negative polyarthritis (RF- polyJIA), rheumatoid factor positive polyarthritis (RF + polyJIA), juvenile psoriatic arthritis (jPsA), enthesitis-related arthritis (ERA-JIA). A seventh category, undifferentiated arthritis (UA), groups cases that don’t fit into any of the other six categories, or that fit into more than one [2]. Other guidelines, however, group several JIA phenotypes together, as genetic evidence suggests that the ILAR categories might not reflect the underlying role of genes and clinical presentation [8]. In the context of this study, polyarticular JIA (polyJIA) will encompass RF- polyJIA, RF + polyJIA, and extended oligoJIA.

Reliable epidemiological figures are imperative for burden of disease estimates and healthcare resource planning. Reported prevalence varies widely depending on geographical location [9], ethnicity [10, 11], JIA category [11–13], and case definition, with higher rates of JIA found in children and adolescents of European descent and in Europe [12]. JIA prevalence estimates in Europe range widely, from 3.8 to 400 per 100,000 [13–16], however more precise and recent prevalence and incidence data is missing from the literature, especially for polyJIA.

Community-based health claims studies use a sample of the population to study epidemiology questions and help fill knowledge gaps [3], but still epidemiological data on a larger, national scale in Germany are scarce. Database studies offer a widespread analysis of the population, however, they rely heavily on case definitions and sample data size and representativeness. To help fill this knowledge gap, we used a more broad range of diagnosis codes than similar study designs [17] thereby improving sensitivity, however defining timing of codes so as to maintain specificity. Applying these criteria to two large health claims databases (covering over 8 million SHI German residents in total), we evaluated prevalence and incidence of JIA, and frequently associated comorbidities of JIA and polyJIA in Germany.

## Methods

### Data source

This was a retrospective, observational cohort study using two different longitudinal health claims databases; the WIG2 (*Wissenschaftliches Institut für Gesundheitsökonomie und Gesundheitssystemforschung*, the Scientific Institute for Health Economics and Health System Research GmbH) and the InGef (*Institut für angewandte Gesundheitsforschung,* Institute for Applied Health Research Berlin GmbH) research databases. A representative sample (in terms of age and gender) of the statutory health insurance (SHI) population per year in Germany from each of the WIG2 (3.5 million patients) [18, 19] and InGef (about 4 million patients) [20–23] research databases were selected for analyses. Each database contains a unique mix of SHI patient data, without overlapping populations.

All healthcare services for which claims are submitted are available in the database (ATC codes, diagnoses, procedure codes) and their respective costs. Demographic data (age and gender), data on outpatient care (diagnoses, procedures, physician specialty, costs), inpatient care (length of stay, procedures, main and secondary diagnoses and reasons for admission and discharge), pharmaceutical data (dispensed drug and quality, and specialty of the prescribing physician), and finally information on medical devices and allied health services (therapy and duration) are available in the databases. For our study objectives, diagnoses (by documented ICD-10 GM, International Classification of Disease, version 10 German modification code) were analysed.

Claims data from both sources are anonymised in a data centre before entering the WIG2 and InGef research databases, removing personal information from patients, healthcare providers (e.g., physicians, clinics, hospitals, pharmacies), and the SHI providing the insurance. Only aggregated data (*n* ≥ 5) can be reported, according to German data protection regulations. As such, no independent ethics committee approval was needed.

### Study design and population

This retrospective observational cohort study analysed data from January 1^st^, 2013 to December 31^st^, 2019 (including baseline and follow-up periods).

Different cohorts were formed (for each of the years 2014 to 2018), by successively applying inclusion criteria to the entire population; patients aged 2–15, with continuous insurance throughout the year and an appropriate JIA diagnosis. Death in the observation year did not result in exclusion. Patients with either at least one main inpatient or two of secondary inpatient and/or confirmed outpatient diagnoses (ICD-10 codes M05, M06, M07, M08, M09, M13, M45, L40.5) in two different quarters within the calendar (index) year (M2Q criterion) met our JIA case definition [24]. Since JIA diagnosis criteria include symptoms that persist for at least 6 weeks [2], other studies have also considered this timeframe in their criteria (depending on the nature of the database) [5, 25]. Since we are only able to identify outpatient diagnoses by quarter, unable to pinpoint a period of exactly 6 weeks between diagnoses, we used M2Q criteria to increase the chances that there were at least 6 weeks between diagnoses. The term polyJIA encompassed the ILAR categories polyarthritis (RF + and RF- polyJIA) and extended oligoJIA in this study, including patients with at least 1 main inpatient discharge diagnosis of ICD-10 codes M08.0 or M08.3 or 2 confirmed outpatient diagnoses of either of M08.0 or M08.3 in different quarters in the respective observation year, with secondary inpatient diagnoses counted as confirmed outpatient diagnoses.

The patients’ JIA category was determined by ICD-10 code (see additional files 1 and 2 for definitions and codes used), and the patient was categorised as having UA if they met the criteria for none of the other, or more than one JIA category, as is intended in the ILAR criteria for UA.

Since children or adolescents occasionally receive adult diagnosis codes [17], we included adult codes in our JIA criteria (in those aged 2–15).

### Outcomes

We calculated prevalence and incidence rates of JIA (overall), polyJIA, and those of all ILAR categories (see additional files 1 and 2 for definitions and codes) for each year, and stratified by age group (2–5 years, 6–11 years, and 12–15 years) and sex (male, female). For incidence rates, patients had to also have continuous insurance in the calendar year prior to the index year, without any documented diagnoses during the 4 quarters prior to the index quarter.

We determined the prevalence of the top 10 most common comorbidities and of particular comorbidities of interest in our prevalent population (stratified into JIA and polyJIA groups); namely the frequencies of allergic rhinitis, predominantly allergic asthma, amyloidosis, anaemia, anxiety disorders, primary hypertension, atopic dermatitis, persistent somatoform pain disorder, ulcerative colitis, depression, diabetes mellitus, iron deficiency, fatigue, fibromyalgia, autoimmune thyroiditis, thyrotoxicosis, hypothyroidism, lack of expected normal physiological development, Crohn’s disease, migraine, kidney disease (chronic), osteoporosis, psoriasis, sicca syndrome (Sjögren’s), and uveitis (see additional files 4, 5, and 6 for ICD-10 codes used).

### Statistical analysis

Prevalence and incidence rates (per 100,000 population) were calculated by dividing the number of patients captured by the criteria by the total number of patients in that age range in the database. Sex-stratified rates were calculated by dividing the number of patients by the absolute number of patients in that age-sex stratum. Using direct standardisation, we extrapolated the prevalence and incidence rates to the entire German population, by age and sex, based on extrapolation statistics of the Federal Statistical Offices [26].

These projections are presented with 95% confidence intervals (95% CIs) to account for uncertainties. The confidence intervals were calculated according to the Fay and Feuer method [27] assuming a Poisson distribution in the data.

The number of cases (n) of pre-defined comorbidities was determined among prevalent JIA and polyJIA populations in 2018, and the numbers were presented as a rate (% with that comorbidity divided by total cases).

### Sensitivity analysis

To assess potential heterogeneity, the data was evaluated on whether estimated prevalence and incidence using the InGef and WIG2 databases (two samples) show an inhomogeneous distribution. To analyse heterogeneity, forest plots were used and a chi-squared homogeneity test was performed. A 99% CI was calculated using normal distribution approximation. Cohen’s d test was used to determine the size of potential differences between both databases.

## Results

**Fig. 1:**
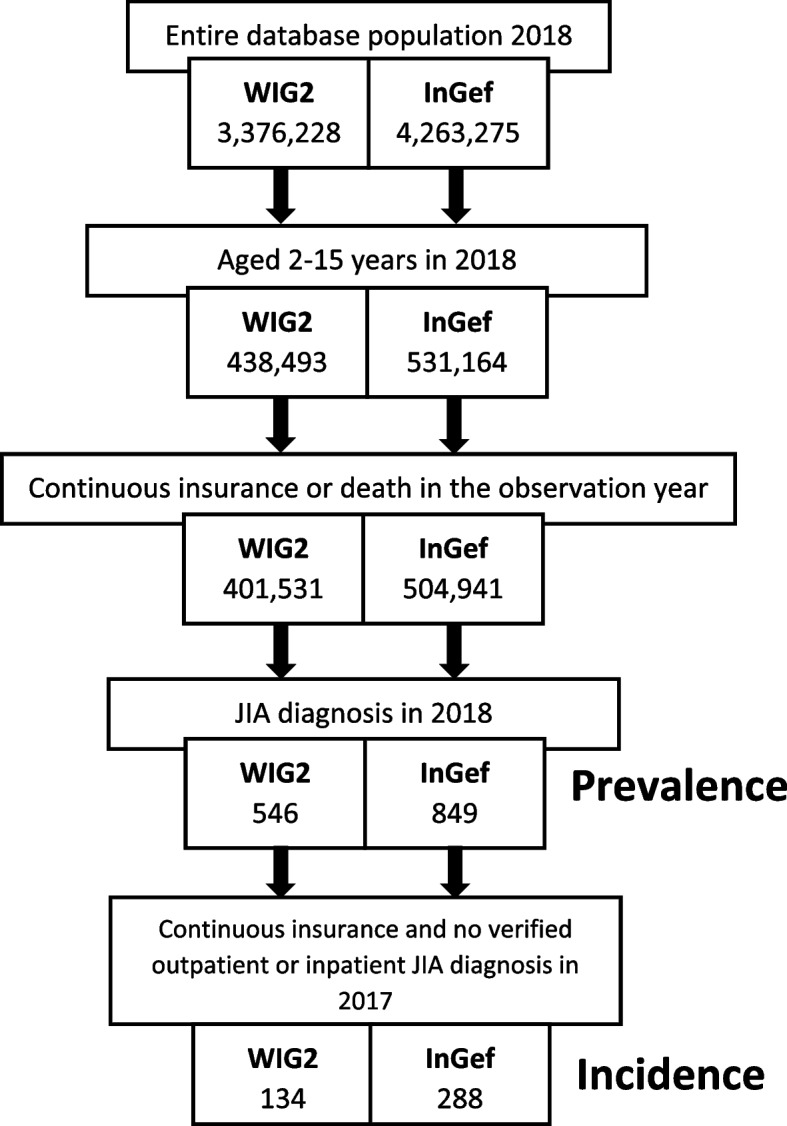
Patient selection of each prevalence and incidence population (data shown for 2018 only)

Of the over 3 and 4 million patients in the WIG2 and InGef databases (respectively) in 2018, 438,493 (13.0%) and 531,164 (12.5%) were aged 2–15 years respectively. Of the 401,531 and 504,941 with continuous insurance in the observation year (WIG2 and InGef databases respectively), 546 and 849 met our JIA definition in 2018. Of these, only 134 and 288 had a year of continuous insurance prior to the observation year with no JIA diagnosis (Fig. 1).

Overall, we found a decreasing trend in JIA prevalence and incidence rates in both databases (see Table 1). In 2018, prevalence among our database patients was 133.21 and 167.76, and incidence was 34.17 and 59.99 per 100,000 German population in the WIG2 and InGef databases.

**Table 1:** Extrapolated overall JIA prevalence and incidence rates per 100,000 German population, with 95% confidence intervals

	**Prevalence**		**Incidence**	
**Year**	WIG2	InGef	WIG2	InGef
**2014**	147.23 (136.50–158.59)	183.45 (171.58–195.93)	51.66 (45.23–58.75)	72.15 (64.63–80.32)
**2015**	139.17 (128.63–150.37)	174.84 (163.38–186.90)	39.11 (33.48–45.44)	62.69 (55.66–70.38)
**2016**	136.95 (126.18–148.40)	176.02 (164.52–188.11)	39.45 (33.62–46.03)	63.67 (56.63–71.35)
**2017**	142.24 (131.08–154.13)	176.26 (164.81–188.31)	43.58 (37.33–50.60)	64.81 (57.75–72.51)
**2018**	133.21 (122.26–144.90)	167.76 (156.66–179.43)	34.17 (28.62–40.51)	59.99 (53.26–67.34)

**Fig. 2:**
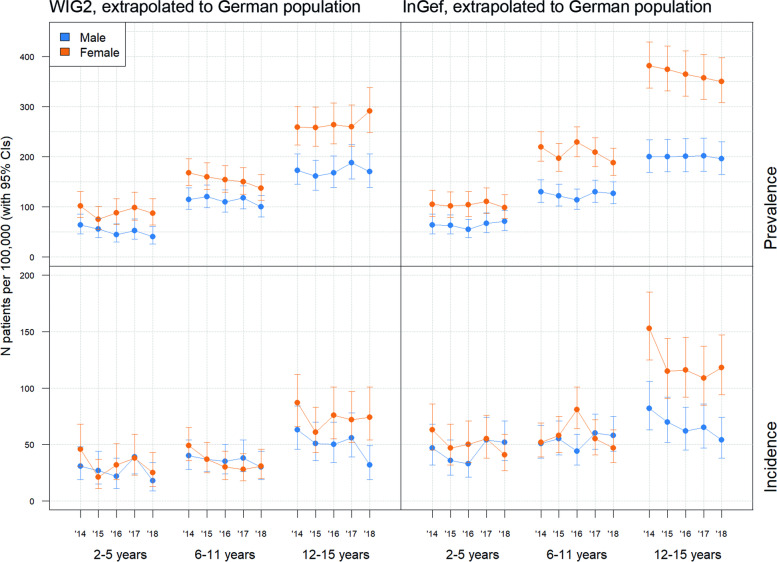
Overall JIA prevalence and incidence rates per 100,000 population in Germany (database data extrapolated). Error bars indicate 95% confidence intervals

We used the data on prevalence and incidence by age, gender, and observation year, and extrapolated this data to the German population (per 100,000 population) (Fig. 2). Over time (from 2014 to 2018) both prevalence and incidence showed a relatively stable pattern, with highs and lows seen in some age/gender groups. In both males and females, there seemed to be a slight overall trend in decreasing prevalence and incidence over the five years.

Both incidence and prevalence were mostly higher in females than in males (Fig. 2). While rates for girls slightly exceeds that of boys in children, the gap between genders appeared to increase with age; in 2018 the per 100,000 population prevalence (with 95% CIs) in females aged 2–5 was 87.39 (63.20–114.42) and 98.61 (77.02–124.39) in the WIG2 and InGef databases respectively, compared to 40.38 (25.59–60.60) and 71.10 (53.41–92.78) in males. However, in females aged 12–15 the same year, it was 290.61 (248.61–338.17) and 350.24 (307.75–396.95) in WIG2 and InGef databases, compared to 170.31 (139.30–206.22) and 195.56 (165.03–230.12) in males (see additional file 3 for complete data). Prevalence was also highest in this group (females aged 12–15), with the lowest prevalence seen in males aged 2–5. The same pattern in incidence rates was observed; females aged 12–15 had higher incidence rates than males in the same age group, and this in both databases. However, the difference in incidence between genders in younger patients seemed to play a smaller role; in fact, in the InGef database, there was more incident disease in males aged 2–5 than in females in 2018, however this was only true in this year (Fig. 2). Otherwise, females were more likely to have incident disease, even in this age group.

The most prevalent JIA category diagnosed was oligoJIA (70 and 91 per 100,000 population), followed by UA (49 and 56 per 100,000 population) and RF-polyJIA (31 and 39 per 100,000) in the WIG2 and InGef databases respectively (Table 2). There were over twice as many patients diagnosed with oligoJIA than RF-polyJIA, and nearly half of all JIA patients had been diagnosed with this category.

**Table 2:** Extrapolated JIA prevalence and incidence (n and rate per 100,000 population in Germany) in 2018 by ILAR category, with 95% confidence intervals

**2018 Prevalence**				
	WIG2		InGef	
**JIA** **ILAR Categories**	n extrapolated (Germany) (95% LCI-RCI) WIG2	Adjusted rates /100,000 (95% LCI-RCI) WIG2	n extrapolated (Germany) (95% LCI-RCI) InGef	Adjusted rates /100,000 (95% LCI-RCI) InGef
**ALL**	13,938 (12,792–15,161)	133 (122–145)	17,552 (16,391–18,774)	168 (157–179)
**1** **sJIA**	924 (647–1283)	9 (6–12)	931 (679–1246)	9 (6–12)
**2** **oligoJIA**	7330 (6503–8236)	70 (62–79)	9495 (8646–10,405)	91 (83–99)
**3** **RF- polyJIA**	3218 (2678–3837)	31 (26–37)	4052 (3504–4661)	39 (33–45)
**4** **RF + polyJIA**	375 (210–624)	4 (2–6)	888 (643–1197)	8 (6–11)
**5** **jPsA**	251 (120–466)	2 (1–4)	579 (384–837)	6 (4–8)
**6** **ERA-JIA**	932 (656–1288)	9 (6–12)	1010 (748–1336)	10 (7–13)
**7** **UA**	5109 (4423–5873)	49 (42–56)	5893 (5228–6619)	56 (50–63)
**2018 Incidence**				
	WIG2		InGef	
**JIA ILAR Categories**	n extrapolated (Germany) (95% LCI-RCI) WIG2	Adjusted rates /100,000 (95% LCI-RCI) WIG2	n extrapolated (Germany) (95% LCI-RCI) InGef	Adjusted rates /100,000 (95% LCI-RCI) InGef
**ALL**	3575 (2995–4239)	34 (29–41)	6277 (5573–7046)	60 (53–67)
**1** **sJIA**	193 (78–402)	2 (1–4)	349 (199–568)	3 (2–5)
**2** **oligoJIA**	1311 (966–1742)	13 (9–17)	2860 (2391–3394)	27 (23–32)
**3** **RF- polyJIA**	472 (275–760)	5 (3–7)	1024 (752–1362)	10 (7–13)
**4** **RF + polyJIA**	-	-	437 (267–675)	4 (3–6)
**5** **jPsA**	-	-	305 (167–513)	3 (2–5)
**6** **ERA-JIA**	339 (181–586)	3 (2–6)	434 (265–671)	4 (3–6)
**7** **UA**	1034 (731–1423)	10 (7–14)	1634 (1285–2049)	16 (12–20)

*Category abbreviations: sJIA* systemic arthritis, *oligoJIA* oligoarthritis, *RF- polyJIA* Rheumatoid factor negative polyarthritis, *RF* + *polyJIA* Rheumatoid factor positive polyarthritis, *jPsA* Psoriatic arthritis, *ERA-JIA* Enthesitis-related arthritis, *UA* Undifferentiated arthritis, *jPsA* Juvenile psoriatic arthritis

The incidence for these three (oligoJIA, UA and RF-polyJIA) in 2018 was also the highest, again with about half of incident JIA patients in 2018 in each database being diagnosed with oligoJIA.

**Fig. 3:**
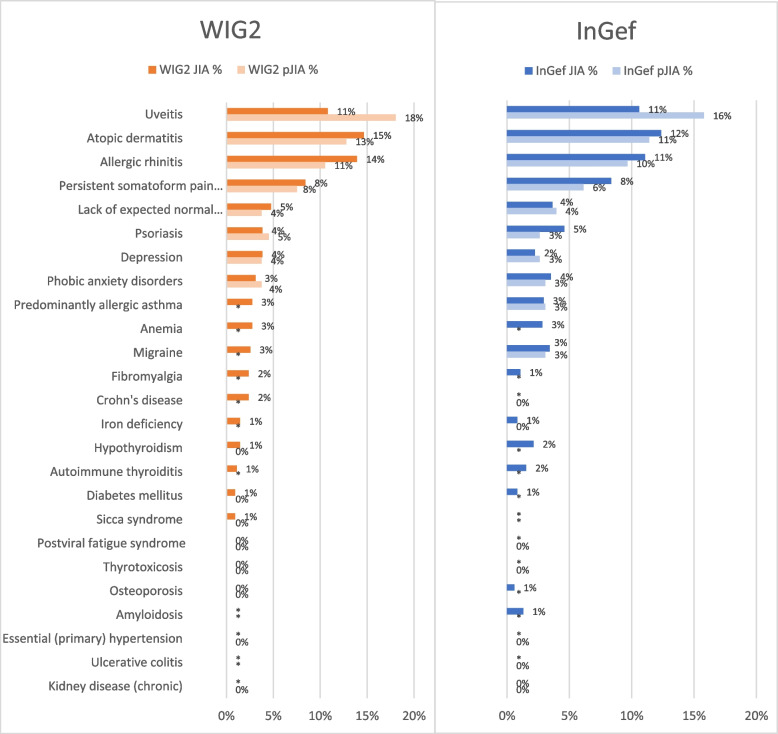
Predefined comorbidities in prevalent JIA and polyJIA patients in 2018 for WIG2 and InGef databases *comorbidities for which the number of cases was < 5 are only reported as such, due to data protection laws. For this reason, a rate could not be generated for these comorbidities

Atopic dermatitis, vasomotor and allergic rhinitis, and uveitis were the pre-defined comorbidities seen most often in both databases, both among patients with prevalent JIA and those with polyJIA, in 2018 (Fig. 3). The top 3 were however ordered differently in patients with JIA in each database; in the WIG2 database, atopic dermatitis was seen most often (14.65%), whereas uveitis was seen more often in the InGef database (16.29%).

The percentage of patients with uveitis was higher in patients categorised as having polyJIA than in the general JIA population (18.05% and 15.79% in each of the WIG2 and InGef databases respectively, compared to 11% of JIA population in both databases). Data on which other comorbidities were seen more often in patients categorised as polyJIA than in the general JIA population were inconsistent between the databases, and the difference was less striking (psoriasis, depression, phobic anxiety disorders, and allergic asthma).

Ulcerative colitis, diabetes mellitus, thyrotoxicosis, kidney disease, and osteoporosis were either not observed at all or the number of patients affected was < 5 (and due to data protection laws not reported precisely) in both JIA and polyJIA patients.

The 10 most common diagnoses observed in prevalent patients of the overall JIA population in both databases (most, but not all diagnoses, overlapped both) were juvenile arthritis, disorders of refraction and accommodation, acute upper respiratory infections, other soft tissue disorders, other joint disorders, other arthritis, abdominal and pelvic pain, other rheumatoid arthritis, viral infections, immunization against viral diseases, other strabismus, general examination, and congenital deformities of feet (see Additional file 4).

## Discussion

Using two large claims databases, we used a broad range of possible JIA-related ICD-10 codes and M2Q diagnosis criteria to screen two large samples of SHI patients in Germany for children and adolescents aged 2 to 15 with JIA.

### Prevalence/incidence

We found overall 2018 JIA prevalence rates of 133.21 and 167.76, and incidence rates of 34.17 and 59.99 per 100,000 German population in the WIG2 and InGef databases, respectively (Fig. 1). These rates are higher than those from another study using SHI claims data, with prevalence rates of 73.4 to 101.5 cases per 100,000 between 2009 and 2015, and incidence rates 16.0 and 17.4 per 100,000 from 2011 to 2015 [17]. The main reason is likely the use of different case definitions; this study excluded inpatient diagnoses and some ICD-10 codes typically used for adults, both of which we included in our analysis (for example codes M05, M06 and M07). For this reason, we would have also captured pubertal adolescents transferring their healthcare to a general practitioner from their paediatrician. Thomschke et al. [17] also excluded patients with an applicable diagnosis in the previous two years for incidence calculations, likely resulting in fewer incident cases than we found by excluding only one year prior, perhaps an indication that our incidence estimation may be an overestimate of true incidence. Another reason for differences could be regional variation; the databases we used were comprised of two different populations for which the patients’ regional composition may have been substantially different, explaining the different prevalence and incidence between our two database populations. Thomschke et al. were able to draw from the entire population treated by SHI-contracted physicians in Germany in that timeframe, and found substantial regional variation depending on the federal state; prevalence ranged from 67.3 to 199.3 per 100,000 children and adolescents in Hessen and Schleswig–Holstein respectively, and incidence ranged from 11.9 to 45.2 new diagnoses per 100,000 population of children and adolescents (in North Rhine-Westphalia and Schleswig–Holstein respectively) [17]. Regional differences in epidemiology have been observed both within and between countries [12, 28], however case definition differences would also play into these differences, perhaps being the most important factor. Prevalence studies going into the community and examining children who have not sought medical attention (and would be overseen in a study like ours) using trained clinicians (thus improving case definition accuracy), do in fact yield higher rates.

As expected, we found higher prevalence rates in females than in males of all ages (Fig. 2), as observed in other database studies [17, 29], and epidemiological data [3]. Female prevalence rates slightly exceeded those of males in pre-pubertal children, with the gap in prevalence between genders increasing with age (and likely with puberty). In contrast, a Taiwanese population study found that more males were affected (female to male ratio was 0.79:1), in their overall JIA population [11], again demonstrating the variance in epidemiological results by geography and ethnicity.

Thomschke et al. [17] found a less substantial difference in both prevalence and incidence between genders, however increases in both with increasing age were also dependent on gender. The higher number of prevalent cases observed in females than males became more distinct with increasing age; in patients aged 0–14 there were 1.89 times more prevalent cases in females than in males, however in ages 15–19 this increased to 2.11 (in 2014). Similar trends were seen in 2014 incidence data; incidence rates were twice as high in females than males (annual average was 21.8 and 11.6 new diagnoses in females and males respectively, per 100,000 female and male children/adolescents, respectively [17]. While our population only included children and adolescents ages 2 to 15, this increasing gap in prevalence and incidence appears to increase beyond this age group; they observed 1.2 times higher incidence in adolescents aged 15–19, compared to children/adolescents aged 0–14 [17]. In contrast to our results of increasing incidence with age, a small regional study on JIA incidence in Sweden found that JIA patients 2 years of age had the highest incidence rate in the study in children < 16 years (36/100,000) [30], however this may have to do with differences in regional awareness of the disease in both the population and the healthcare system.

An evaluation of a 2018 InGef database sample for a benefit assessment of tofacitinib [31] evaluated polyJIA epidemiology using very similar criteria as our study found a prevalence rate of 46.08 cases per 100,000 children and adolescents aged 2–17, and incidence 12.75 cases per 100,000 children and adolescents aged 2–15. We observed similar gender and age trends in our overall JIA patients, to those in their polyJIA population [31]; other studies report similar gender patterns in some ILAR categories (oligoJIA and polyarticular JIA) [3, 13], those of which comprised most of our population (see Table 2).

### ILAR categories

The highest proportion of our JIA patients in 2018 had received a diagnosis categorised as oligoJIA (70 and 91 cases, from a total of 122 and 168 JIA cases, per 100,000 population in the WIG2 and InGef databases respectively) (Table 2), which has also been observed in pooled results from various studies [15].

This is also in line with a recent German inception cohort of about 953 JIA patients newly diagnosed in 10 German tertiary centres of whom 45.8% were classified as having oligoJIA followed by 26.2% with RF- polyJIA, 10.5% with ERA-JIA, 1.5% with RF + polyJIA, 4.2% with jPsA, 3.7 with sJIA, and finally 7.5% with UA [32].

The second highest proportion of our JIA patients with incident disease in 2018 were categorised as having undifferentiated arthritis (UA) (10 and 16 per 100,000 population in Germany, from WIG2 and InGef databases respectively), meaning they fit into either none of the other, or two or more, ILAR categories. This may indicate either uncertainty in diagnosis or the need for improved description or diagnostic criteria of JIA subcategories. One example is the presence of HLA-B27 in a boy, which occurs in about 10% of all patients but is an exclusion criterion for all other JIA categories but ERA-JIA. Despite the presence of psoriasis or polyarthritis and rheumatoid factor, those patients must be labelled as UA and therefore more than 10% unclassifiable patients are expected. Our understanding of the molecular basis of JIA categories is still unfolding and debate and evaluation of classification is by no means final [33]. It is possible that, for some patients, the category UA is initially chosen, with another category being specified after some time observing the course of the disease, since diagnosis appears to take more than one visit to a physician; a small French study found an average of three physician visits took place and that over half the patients waited an average median time of three months for a diagnosis, with the diagnosis often being made by paediatric rheumatologists [34]. The study found that patients with sJIA, ERA-JIA, oligoJIA and polyJIA were diagnosed immediately upon presentation. However, due to a similarly large proportion of patients falling into this category observed also in prevalence data (49 and 56 per 100,000 population in Germany, from WIG2 and InGef databases, respectively), it appears unlikely that further specification into another ILAR category occurred.

These results are in contrast with other literature; a study in Turkey (representing another geographic and ethnic study population) in a relatively small study of 198 patients with JIA found that the largest proportion of patients (26.3%) had been diagnosed with sJIA, followed by oligoJIA (18.7%) and RF– polyarthritis (17.2%) [35].

A Taiwanese study evaluated the risk of malignancy in JIA patients, and stratified 2892 JIA patients by ILAR category (using diagnosis codes). In this study, the only ILAR categories used were sJIA, polyarthritis, pauciarthritis or oligoarthritis, ERA and jPsA [11]. They found the largest group to have ERA (42% of all JIA patients), followed closely by unclassifiable (38%). In contrast, we saw the highest proportion of patients fall into the oligoJIA category, though this was also followed by undifferentiated arthritis.

### Comorbidities

The top 10 comorbidities among our prevalent JIA population were as could be expected in our population; the most frequently observed extraarticular diagnoses were uveitis, followed by atopic dermatitis and rhinitis, the latter expected in patients in this age group (Fig. 3). Of notice, pain disorders, impaired or lack of development, depression and phobic/anxiety disorders were 4 of the 5 following disorders next to them and highlighting the importance of mental health in chronically ill children [36].

We found uveitis documented in 10% and 16% of JIA patients, and 18% and 16% of polyJIA patients in the WIG2 and InGef databases, respectively (Fig. 3). This is similar to results observed in international studies (10% in a small study in Turkey and 13.1% in a large rheumatology database in Canada) [35, 37]. The two other most frequently observed comorbidities (allergic rhinitis and atopic dermatitis) appear to be seen at higher rates in our JIA and pJIA populations, as they are in the general paediatric population in Germany [38, 39].

While the association of some comorbidities (particularly other autoimmune diseases, [40]) with a JIA diagnosis has been demonstrated in previous studies, whether they are associated with the disease aetiology or other factors, such as treatment, remains unclear [41]; the BIKER registry evaluates tolerance of different treatments, and their side effects and efficacy, showing that certain associated conditions (especially infections, such as herpes zoster) are associated more highly with particular treatments [42]. Some treatments however seem to demonstrate a protective effect against some side effects of JIA; new occurrence of uveitis was observed in patients treated with etanercept and methotrexate, but not in those treated with adalimumab, suggesting the latter may have a protective effect. Due to the short observation time in the registry, however, this was not conclusive [42].

Less than 1% of patients had a Crohn’s disease diagnosis in each of our databases. There were some polyJIA cases in the WIG2 database with ulcerative colitis and Crohn’s disease, also with case numbers too small to report. A study looking at inflammatory bowel disease (IBD) in biologic naïve patients with JIA found that IBD occurrence was possibly tied to the type of treatment initiated; IBD incidence was significantly lower in patients treated with methotrexate, but significantly higher in patients treated with etanercept or sulfasalazine [41]. According to recent German data on the age-adjusted incidence rate of IBD in children and adolescents under 15 years of age at diagnosis [43], there were 6.1 (95% CI 3.7–8.4) cases of Crohn’s disease per 100,000 person years (PYs) in 2009, which is higher than the rate we observed in our WIG2 database.

We found 4.76% and 4.55% of patients with JIA (WIG2 and InGef databases respectively) and 3.76% and 3.95% of polyJIA patients (in WIG2 and InGef databases respectively) had documented lack of expected normal physiological development. Giannini et al. [44] discuss different growth failure rates by JIA category, affecting about 10% and 41% of patients with polyarticular and systemic arthritis categories respectively.

We found different results between the two databases; while the InGef database had a larger database sample (4,263,275 compared to 3,376,228 in the WIG2 database) (Fig. 1) and therefore more absolute prevalent and incident cases, the rate per 100,000 population was still higher in data from the InGef database. The difference in results between the two databases can be partly explained by the nature of this relatively rare disease; even small differences in these large samples have a large impact and can yield statistically significant differences. Using Cohen’s d test, we determined that these statistically significant differences are minimally relevant (< 0.01).

While our populations are representative of the German population in terms of age and sex, representativeness of other factors, such as geographic location, socioeconomic status or ethnicity cannot be guaranteed, all of which may affect JIA prevalence [10, 11, 45]. Regional differences in coding habits of physicians may occur, perhaps due to selective contracts or different training in physicians by region, possibly having a large impact on a rare disease often diagnosed by specialists. If any of the outcomes are influenced by these factors, the generalisability of our results on a national scale would also be impacted.

#### Potential limitations

The analyses are based on data collected for billing purposes and not for epidemiological studies. There is potential for errors in billing and in data entry or coding; for example, diagnoses for patients are coded by healthcare providers that do not entirely correspond to the clinical picture of the patient. Also, only patients who actually sought medical care or advice in the given timeframe were analysed. Otherwise they would not appear in our database; community studies which evaluated children often found higher JIA rates than those only evaluating patients who sought medical attention for related symptoms (25, 26).

This potential limitation has been addressed by including restrictive definitions based on inpatient diagnoses and confirmed outpatient diagnoses in combination with prescriptions typical for the clinical picture. It is therefore assumed that the inclusion of false-positive cases was comparatively low.

Claims data has been found to identify JIA patients with a reliability of 81–86% using similar, if not slightly less stringent criteria than the criteria we used (2 diagnoses within 2 years, at least 8 weeks apart) [25]. Since our cohorts included patients according to the M2Q criteria, a relatively conservative estimation, our patient population would have been limited to patients with these criteria within the calendar year and would have excluded patients with the same diagnoses that extend either into the following year, or from the previous year (for prevalent patients). Studies which capture JIA cases with only one confirmed outpatient diagnosis almost always have higher case numbers and may capture a large number of cases that are not confirmed as JIA after further review [30]. Furthermore, we included patients for whom only one inpatient diagnosis was documented, without the minimum of 6 weeks between diagnosis codes; these patients may not fulfil this aspect of diagnosis criteria [2].

Our incidence calculations were based on a year of baseline prior to the first observed diagnosis, which means any diagnoses that occurred prior to this baseline year were not considered, resulting in some uncertainty in actual incidence data.

The research databases used for the analyses are representative of the German SHI population in terms of age and sex [23], however regional representation is not guaranteed. The two databases we used may have different regional representation, and region has been observed in other studies to play a role in JIA prevalence rates [17]. The extent of missing data (incomplete insurance) is very small and the impact on the analyses is therefore negligible. A benefit, however, is that billing data is not affected by health care providers or patients’ willingness to participate. Since consent is not required (and data is anonymous), data protection laws require that measures are in place to ensure patients cannot be identified, by requiring categories for which the patient number is less than 5 be identified only as such. This plays an important role in our study, since case numbers of rare diseases such as JIA, and especially its categories, often resulted in case numbers of less than 5.

Health insurance billing data do not allow classification of socioeconomic status (income, education, occupation), so this determinant could not be considered. Descriptive analyses based on health insurance data were performed. Statements on causal relationships are not possible with the results.

## Conclusion

This study presents the most current data on prevalence and incidence in Germany, using a different mix of ICD-10 codes from healthcare data to improve sensitivity, while still maintaining specificity in results using M2Q criteria. Using our definition, we found higher prevalence and incidence rates than other database studies in Germany, however community-based evaluations appear to remain more sensitive, typically resulting in higher rates.

Female prevalence rates slightly exceeded those of males in pre-pubertal children, with the gap in prevalence between genders increasing with age, and more than half of our JIA patients had received a diagnosis categorised as oligoJIA.

We evaluated differences in comorbidities between JIA (overall) and categories of polyJIA patients and found that uveitis was documented more often in polyJIA patients.

Our results help enrich the knowledge on epidemiology, thereby contributing to the comprehension of the disease and its management.

## Supplementary Information

**Additional file 1. **ICD-10 codes used to identify JIA in the databases.

**Additional file 2. **ILAR categories defined, and ICD-10 codes used to identify them.

**Additional file 3. **JIA prevalence and incidence rates by age group, extrapolated to population in Germany (per 100,000), with 95% confidence intervals.

**Additional file 4. **Frequency (n and rate in %) of the 10 most commonly coded ICD-10 codes among prevalent JIA patients overall in 2018, for both WIG2 and InGef databases.

**Additional file 5. **Frequency (n, and rate in %) of predefined comorbidities in prevalent JIA patients overall in 2018, for each of WIG2 and InGef databases.

**Additional file 6. **Frequency (n and rate in %) of pre-defined comorbidities in prevalent polyarticular JIA patients overall in 2018 for each of WIG2 and InGef databases.

## Data Availability

The datasets generated and analysed during the current study are not publicly available due to data protection laws. Raw dataset data are not publicly available to preserve individuals’ privacy under the European General Data Protection Regulation.

## Abbreviations

bDMARDs: Biological disease-modifying anti-rheumatic drugs
csDMARDs: Conventional synthetic disease-modifying anti-rheumatic drugs
ERA: Enthesitis-related arthritis
GC: Glucocorticoids
IBD: Inflammatory bowel disease
ICD-10 GM: International Classification of Disease, version 10 German modification
ILAR: International League of Associations for Rheumatology
InGef: *Institut für angewandte Gesundheitsforschung,* Institute for Applied Health Research Berlin GmbH
JAK: Janus kinase
JIA: Juvenile idiopathic arthritis
NSAID: Non-steroidal anti-inflammatory drugs
oligoJIA: Oligoarthritis
polyJIA: Polyarticular juvenile idiopathic arthritis
jPsA: Juvenile psoriatic arthritis
RF + polyJIA: Rheumatoid factor positive polyarthritis
RF-polyJIA: Rheumatoid factor negative polyarthritis
SHI: Statutory health insurance
sJIA: Systemic juvenile idiopathic arthritis
UA: Undifferentiated arthritis
WIG2: *Wissenschaftliches Institut für Gesundheitsökonomie und Gesundheitssystemforschung* The Scientific Institutefor Health Economics and Health System Research (WIG2 GmbH)
