# Supplementary material for: Incidence, prevalence, and comorbidities of juvenile idiopathic arthritis in Germany: a retrospective observational cohort health claims database study
Source: Pediatr Rheumatol Online J. 2022 Nov 16;20:100. doi: 10.1186/s12969-022-00755-x (PMC9670409; doi:10.1186/s12969-022-00755-x)
Supplement: Supplementary file 1 — Additional file 1. ICD-10 codes used to identify JIA in the databases. [file 12969_2022_755_MOESM1_ESM.docx]

[Additional file 1] ICD-10 codes used to identify JIA in the databases

| **ICD-10 diagnostic code (German version)** | | English translation | |
| --- | --- | --- | --- |
| M05.- Seropositive chronische Polyarthritis | | Rheumatoid factor positive rheumatoid arthritis | |
| M06.- Sonstige chronische Polyarthritis | | Other rheumatoid arthritis | |
| M07.- Arthritis psoriatica und Arthritiden bei gastrointestinalen Grundkrankheiten | | Psoriatic and enteropathic arthropathies | |
| M08.- Juvenile Arthritis | | Juvenile arthritis | |
| M09.- Juvenile Arthritis bei anderenorts klassifizierten Krankheiten | | Juvenile arthritis in diseases classified elsewhere | |
| M13.- Sonstige Arthritis | | Other arthritis | |
| M45.- Spondylitis ankylosans | | Ankylosing spondylitis | |
| L40.5+ Psoriasis-Arthropathie (M07.0-M07.3*)(M09.0-*) | | Arthropathic psoriasis | |
