# Supplementary material for: Incidence, prevalence, and comorbidities of juvenile idiopathic arthritis in Germany: a retrospective observational cohort health claims database study
Source: Pediatr Rheumatol Online J. 2022 Nov 16;20:100. doi: 10.1186/s12969-022-00755-x (PMC9670409; doi:10.1186/s12969-022-00755-x)
Supplement: Supplementary file 2 — Additional file 2. ILAR categories defined, and ICD-10 codes used to identify them. [file 12969_2022_755_MOESM2_ESM.docx]

[Addition file 2] ILAR categories defined, and ICD-10 codes used to identify them

| **ILAR subtype** | **Definition** | **ICD-10 code** |
| --- | --- | --- |
| 1. **Systemic Arthritis (sJIA)** | Arthritis in 1 or more joints with or preceded by fever of at least 2 weeks’ duration which is documented to be daily (“quotidian”) for at least 3 days, and accompanied by 1 or more of the following:   - Evanescent (nonfixed) erythematous rash - Generalized lymph node enlargement - Hepatomegaly and/or splenomegaly - Serositis | M08.2 |
| 1. **Oligoarthritis (OA)**    - **Persistent**    - **Extended** | Arthritis affecting 1 to 4 joints during the first 6 months of disease.   - Affecting not more than 4 joints throughout the disease course - Affecting a total of more than 4 joints after the first 6 months of disease | M08.4   M08.3 |
| 1. **Polyarthritis with Rheumatoid Factor Negative (RF- polyJIA)** | Arthritis affecting 5 or more joints during the first 6 months of disease; a test for RF is negative | M08.3 |
| 1. **Polyarthritis with Rheumatoid Factor Positive (RF+ polyJIA)** | Arthritis affecting 5 or more joints during the first 6 months of disease; 2 or more tests for RF at least 3 months apart during the first 6 months of disease are positive. | M08.0 |
| 1. **Psoriatic Arthritis (jPsA)** | Arthritis and psoriasis, or arthritis and at least 2 of the following:   - Dactylitis - Nail pitting or onycholysis - Psoriasis in a first-degree relative | L40.5 |
| 1. **Enthesitis Related Arthritis (ERA)** | Arthritis and enthesitis, or arthritis or enthesitis with at least 2 of the following:   - The presence or a history of sacroiliac joint tenderness and/or inflammatory lumbosacral pain - The presence of HLA-B27 antigen - Onset of arthritis in a male over 6 years of age - Acute (symptomatic) anterior uveitis - History of ankylosing spondylitis, enthesitis related arthritis, sacroiliitis with inflammatory bowel disease, Reiter’s syndrome, or acute anterior uveitis in a first-degree relative | M08.89  M45.-  M08.1 |
| 1. **Undifferentiated Arthritis (UA)** | Arthritis that fulfils criteria in no other subtype or in 2 or more of the above subtypes. | M08.9 |
