# Supplementary material for: Incidence, prevalence, and comorbidities of juvenile idiopathic arthritis in Germany: a retrospective observational cohort health claims database study
Source: Pediatr Rheumatol Online J. 2022 Nov 16;20:100. doi: 10.1186/s12969-022-00755-x (PMC9670409; doi:10.1186/s12969-022-00755-x)
Supplement: Supplementary file 3 — Additional file 3. JIA prevalence and incidence rates by age group, extrapolated to population in Germany (per 100,000), with 95% confidence intervals. [file 12969_2022_755_MOESM3_ESM.docx]

[Additional file 3] JIA prevalence and incidence rates by age group, extrapolated to population in Germany (per 100,000), with 95% confidence intervals

|  |  |  | Prevalence | | Incidence | |
| --- | --- | --- | --- | --- | --- | --- |
|  |  |  | WIG2 | InGef | WIG2 | InGef |
| 2-5 | M | 2014  2015  2016  2017  2018 | 63.85 (46.02-86.39)  55.83 (39.10-77.42)  44.95 (29.61-65.49)  53.34 (36.24-75.75)  40.38 (25.59-60.60) | 64.12 (46.40-86.39)  62.86 (45.68-84.39)  54.71 (38.90-74.80)  66.75 (49.38-88.26)  71.10 (53.41-92.78) | 30.90 (18.60-48.36)  26.81(15.32-43.69)  21.70 (11.21-38.02)  38.70 (23.95-59.21)  18.66 (8.95-34.35) | 47.36 (31.95-67.62)  36.03 (22.84-54.11)  32.7 (20.52-49.63)  53.55 (37.70-73.86)  51.57 (36.31-71.12) |
|  | F | 2014  2015  2016  2017  2018 | 102.41 (78.71-130.56)  75.34 (55.05-100.35)  87.60 (64.56-114.77)  98.86 (74.23-128.33)  87.39 (63.20-114.42) | 105.07(81.42-133.44)  102.41 (79.53-129.84)  103.79 (80.91-131.15)  110.69 (87.36-138.35)  98.61 (77.02-124.39) | 46.29 (30.50-67.39)  21.35 (11.03-37.33)  31.85 (18.55-51.14)  38.45 (23.48-59.45)  25.11 (13.37-43.00) | 62.94 (44.54-86.41)  47.45 (31.78-68.20)  50.20 (34.33-70.92)  54.69 (38.30-75.77)  41.07 (27.29-59.4) |
| 6-11 | M | 2014  2015  2016  2017  2018 | 115.17 (95.25-138.06)  120.20 (99.57-143.86)  110.01 (89.79-133.43)  117.73 (96.48-142.28)  100.07 (80.26-123.36) | 129.98 (109.05-153.76)  122.10 (102.09-144.90)  113.78 (94.49-135.86)  129.65 (109.06-153.00)  126.97 (106.74-149.92) | 39.53 (27.97-54.29)  37.20 (25.91-51.76)  34.98 (23.76-49.68)  38.25 (26.33-53.75)  29.73 (19.24-43.98) | 50.75 (37.79-66.75)  54.49 (40.93-71.12)  43.75 (31.79-58.76)  59.93 (45.84-77.00)  57.91 (44.19-74.56) |
|  | F | 2014  2015  2016  2017  2018 | 167.89 (142.76-195.68)  160.09 (135.29-187.60)  153.91 (128.87-181.70)  149.73 (124.76-177.83)  137.44 (63.20-114.42) | 218.48 (190.38-249.58)  197.30 (170.90-226.63)  228.44 (199.99-259.82)  208.25 (181.22-238.19)  188.39 (162.88-216.78) | 48.93 (35.69-65.52)  36.93 (25.42-51.89)  29.76 (19.26-43.95)  28.09 (17.81-42.19)  31.38 (20.30-46.38) | 52.39 (38.88-69.08)  57.51 (43.20-75.07)  80.69 (63.68-100.87)  55.05 (41.24-72.03)  47.01 (34.41-62.72) |
| 12-15 | M | 2014  2015  2016  2017  2018 | 173.12 (144.64-205.59)  160.79 (132.98-192.72)  168.01 (138.66-201.96)  187.79 (155.82-224.44)  170.31 (139.30-206.22) | 199.55 (168.99-234.04)  200.37 (169.88-234.76)  201.00 (170.22-235.75)  201.56 (170.60-236.53)  195.56 (165.03-230.12) | 62.15 (45.33-83.19)  51.42 (36.01-71.20)  49.20 (33.65-69.50)  56.40 (39.28-78.50)  31.59 (19.02-49.42) | 82.14 (62.68-105.74)  70.12 (52.04-92.45)  61.86 (44.95-83.05)  64.79 (47.43-86.42)  53.75 (38.03-73.78) |
|  | F | 2014  2015  2016  2017  2018 | 259.33 (222.72-299.22)  257.88 (220.69-298.27)  264.16 (225.27-306.67)  259.56 (220.43-303.07)  290.61 (248.61-338.17) | 380.85 (337.08-428.72)  373.67 (330.35-421.10)  363.59 (320.49-410.87)  357.29 (314.45-404.34)  350.24 (307.75-396.95) | 86.79 (66.07-111.97)  60.74 (43.40-82.740)  75.49 (55.27-100.73)  71.47 (51.51-96.67)  74.64 (53.79-100.97) | 152.44 (124.80-184.37)  115.11 (90.99-143.67)  116.30 (91.93-145.15)  108.88 (85.35-136.91)  118.24 (93.61-147.37) |
| Total |  | 2014  2015  2016  2017  2018 | 147.23 (136.50-158.59)  139.17 (128.63-150.37)  136.95 (126.18-148.40)  142.24 (131.08-154.13)  133.21 (122.26-144.90) | 183.45 (171.58-195.93)  174.84 (163.38-186.90)  176.02 (164.52-188.11)  176.26 (164.81-188.31)  167.76 (156.66-179.43) | 51.66 (45.23-58-75)  39.11 (33.48-45.44)  39.45 (33.62-46.03)  43.58 (37.33-50.60)  34.17 (28.62-40.51) | 72.15 (64.63-80.32)  62.69 (55.66-70.38)  63.67 (56.63-71-35)  64.81 (57.75-72.51)  59.99 (53.26-67.34) |
