# Supplementary material for: Incidence, prevalence, and comorbidities of juvenile idiopathic arthritis in Germany: a retrospective observational cohort health claims database study
Source: Pediatr Rheumatol Online J. 2022 Nov 16;20:100. doi: 10.1186/s12969-022-00755-x (PMC9670409; doi:10.1186/s12969-022-00755-x)
Supplement: Supplementary file 4 — Additional file 4. Frequency (n and rate in %) of the 10 most commonly coded ICD-10 codes among prevalent JIA patients overall in 2018, for both WIG2 and InGef databases. [file 12969_2022_755_MOESM4_ESM.docx]

[Additional file 4] Frequency (n and rate in %) of the 10 most commonly coded ICD-10 codes among prevalent JIA patients overall in 2018, for both WIG2 and InGef databases

|  |  | WIG2 | | InGef | |
| --- | --- | --- | --- | --- | --- |
| ICD-10 code | Explanation | N (2018) | Rate | N (2018) | Rate |
| M08 | Juvenile Arthritis | 435 | 79.67 | 689 | 81.15 |
| H52 | Disorders of refraction and accommodation | 280 | 51.28 | 411 | 48.41 |
| J06 | Acute upper respiratory infections of multiple and unspecified sites | 232 | 42.49 | 331 | 38.99 |
| M79 | Other soft tissue disorders, not elsewhere classified | 205 | 37.55 | 293 | 34.51 |
| M25 | Other joint disorders, not elsewhere classified | 191 | 34.98 | 309 | 36.4 |
| M13 | Other arthritis | 188 | 34.43 | 295 | 34.75 |
| R10 | Abdominal and pelvic pain | 108 | 19.78 | 139 | 16.37 |
| M06 | Other rheumatoid arthritis | 107 | 19.60 | 138 | 16.25 |
| B34 | Viral infection of unspecified site | 104 | 19.05 | 145 | 17.08 |
| Z25 | Need for immunization against other single viral diseases | 101 | 18.50 | 145 | 17.08 |
| H50 | Other strabismus | 101 | 18.50 | 172 | 20.26 |
| Z00 | General examination and investigation | 97 | 17.77 | 235 | 27.68 |
| Q66 | Congenital deformities of feet | 91 | 16.67 | 150 | 17.67 |
