# Supplementary material for: Incidence, prevalence, and comorbidities of juvenile idiopathic arthritis in Germany: a retrospective observational cohort health claims database study
Source: Pediatr Rheumatol Online J. 2022 Nov 16;20:100. doi: 10.1186/s12969-022-00755-x (PMC9670409; doi:10.1186/s12969-022-00755-x)
Supplement: Supplementary file 5 — Additional file 5. Frequency (n, and rate in %) of predefined comorbidities in prevalent JIA patients overall in 2018, for each of WIG2 and InGef databases. [file 12969_2022_755_MOESM5_ESM.docx]

[Additional file 5] Frequency (n, and rate in %) of predefined comorbidities in prevalent JIA patients overall in 2018, for each of WIG2 and InGef databases

|  |  | WIG2 | | InGef | |
| --- | --- | --- | --- | --- | --- |
| ICD-10 code | Explanation | N (2018) | Rate | N (2018) | Rate |
| J30.1, J30.2, J30.3, J30.4 | Allergic rhinitis | 76 | 13.92 | 94 | 11.07 |
| J45.0 | Predominantly allergic asthma | 15 | 2.75 | 25 | 2.94 |
| E85 | Amyloidosis | <5 | - | 11 | 1.3 |
| D50, D51, D52, D53, D63, D64 | Anaemia | 15 | 2.75 | 24 | 2.83 |
| F40, F41 | Phobic anxiety disorders | 17 | 3.11 | 30 | 3.53 |
| I10 | Essential (primary) hypertension | <5 | - | <5 | <5 |
| L20 | Atopic dermatitis | 80 | 14.65 | 105 | 12.37 |
| F45.4, R52 | Persistent somatoform pain disorder | 46 | 8.42 | 71 | 8.36 |
| K51 | Ulcerative colitis | <5 | - | <5 | <5 |
| F32, F33, F34, F38.1 | Depression | 21 | 3.85 | 19 | 2.24 |
| E10, E11, E12, E13, E14 | Diabetes mellitus | 5 | 0.92 | 7 | 0.82 |
| E61.1, D50.0, D50.1, D50.8 | Iron deficiency | 8 | 1.47 | 7 | 0.82 |
| G93.3 | Postviral fatigue syndrome | 0 | 0.00 | <5 | <5 |
| M79.7 | Fibromyalgia | 13 | 2.38 | 9 | 1.06 |
| E06.3 | Autoimmune thyroiditis | 6 | 1.10 | 13 | 1.53 |
| E05 | Thyrotoxicosis | 0 | 0.00 | <5 | <5 |
| E00, E01, E03 | Hypothyroidism | 8 | 1.47 | 18 | 2.12 |
| R62, E34.3, E45 | Lack of expected normal physiological development | 26 | 4.76 | 31 | 3.65 |
| K50 | Crohn‘s disease | 12 | 2.20 | <5 | <5 |
| G43, G44.0, G44.2 | Migraine | 14 | 2.56 | 29 | 3.42 |
| N18, N19 | Kidney disease (chronic) | <5 | - | 0 | 0 |
| M80, M81, M82 | Osteoporosis | 0 | 0.00 | 5 | 0.59 |
| L40 | Psoriasis | 21 | 3.85 | 39 | 4.59 |
| M35.0 | Sicca syndrome | 5 | 0.92 | <5 | <5 |
| H20.0, H20.1, H20.2, H20.9, H30.2, H22.0*, H22.1*, H44.1, B00.5 | Uveitis | 59 | 10.81 | 90 | 10.6 |
