# Supplementary material for: Incidence, prevalence, and comorbidities of juvenile idiopathic arthritis in Germany: a retrospective observational cohort health claims database study
Source: Pediatr Rheumatol Online J. 2022 Nov 16;20:100. doi: 10.1186/s12969-022-00755-x (PMC9670409; doi:10.1186/s12969-022-00755-x)
Supplement: Supplementary file 6 — Additional file 6. Frequency (n and rate in %) of pre-defined comorbidities in prevalent polyarticular JIA patients overall in 2018 for each of WIG2 and InGef databases. [file 12969_2022_755_MOESM6_ESM.docx]

[Additional file 6] Frequency (n and rate in %) of pre-defined comorbidities in prevalent polyarticular JIA patients overall in 2018 for each of WIG2 and InGef databases.

|  |  | WIG2 | | InGef | |
| --- | --- | --- | --- | --- | --- |
| ICD-10 code | Explanation | N (2018) | rate | N (2018) | rate |
| Allergic rhinitis | J30.1, J30.2, J30.3, J30.4 | 14 | 10.53% | 22 | 9.65% |
| Predominantly allergic asthma | J45.0 | <5 | - | 7 | 3.07% |
| Amyloidosis | E85 | <5 | - | <5 | - |
| Anemia | D50, D51, D52, D53, D63, D64 | <5 | - | <5 | - |
| Phobic anxiety disorders | F40, F41 | 5 | 3.76% | 7 | 3.07% |
| Essential (primary) hypertension | I10 | 0 | 0.00% | 0 | 0.00% |
| Atopic dermatitis | L20 | 17 | 12.78% | 26 | 11.4% |
| Persistent somatoform pain disorder | F45.4, R52 | 10 | 7.52% | 14 | 6.14% |
| Ulcerative colitis | K51 | <5 | - | 0 | 0.00% |
| Depression | F32, F33, F34, F38.1 | 5 | 3.76% | 6 | 2.63% |
| Diabetes mellitus | E10, E11, E12, E13, E14 | 0 | 0.00% | <5 | - |
| Iron deficiency | E61.1, D50.0, D50.1, D50.8 | <5 | - | 0 | 0.00% |
| Postviral fatigue syndrome | G93.3 | 0 | 0.00% | 0 | 0.00% |
| Fibromyalgia | M79.7 | <5 | - | <5 | - |
| Autoimmune thyroiditis | E06.3 | <5 | - | <5 | - |
| Thyrotoxicosis | E05 | 0 | 0.00% | 0 | 0.00% |
| Hypothyroidism | E00, E01, E03 | 0 | 0.00% | <5 | - |
| Lack of expected normal physiological development | R62, E34.3, E45 | 5 | 3.76% | 9 | 3.95% |
| Crohn’s disease | K50 | <5 | - | 0 | 0.00% |
| Migraine | G43, G44.0, G44.2 | <5 | - | 7 | 3.07% |
| Kidney disease (chronic) | N18, N19 | 0 | 0.00% | 0 | 0.00% |
| Osteoporosis | M80, M81, M82 | 0 | 0.00% | <5 | - |
| Psoriasis | L40 | 6 | 4.51% | 6 | 2.63% |
| Sicca syndrome | M35.0 | 0 | 0.00% | <5 | - |
| Uveitis | H20.0, H20.1, H20.2, H20.9, H30.2, H22.0*, H22.1*, H44.1, B00.5 | 24 | 18.05% | 36 | 15.79% |
